# Ischemia-Induced Cognitive Impairment Is Improved via Remyelination and Restoration of Synaptic Density in the Hippocampus after Treatment with COG-Up^®^ in a Gerbil Model of Ischemic Stroke

**DOI:** 10.3390/vetsci8120321

**Published:** 2021-12-10

**Authors:** Tae-Kyeong Lee, Junkee Hong, Ji-Won Lee, Sung-Su Kim, Hyejin Sim, Jae-Chul Lee, Dae Won Kim, Soon Sung Lim, Il Jun Kang, Moo-Ho Won

**Affiliations:** 1 Department of Food Science and Nutrition, Hallym University, Chuncheon 24252, Korea; tk_lee@hallym.ac.kr (T.-K.L.); limss@hallym.ac.kr (S.S.L.); 2 Department of Global Innovative Drug, Chung-Ang University, Seoul 06974, Korea; jk.hong@famenity.com; 3 Famenity Co., Ltd., Uiwang 16006, Korea; jiwon.lee@famenity.com (J.-W.L.); sungsu.kim@famenity.com (S.-S.K.); 4 Department of Neurobiology, School of Medicine, Kangwon National University, Chuncheon 24341, Korea; janny20@naver.com (H.S.); anajclee@kangwon.ac.kr (J.-C.L.); 5 Department of Biochemistry and Molecular Biology, Research Institute of Oral Sciences, College of Dentistry, Kangnung-Wonju National University, Gangneung 25457, Korea; kimdw@gwnu.ac.kr

**Keywords:** Cornu Ammonis 1, glutaminergic synapse, ischemia-reperfusion injury, memory function, oligodendrocyte, pyramidal cells

## Abstract

Cerebrovascular disease such as ischemic stroke develops cognitive impairment due to brain tissue damage including neural loss, demyelination and decrease in synaptic density. In the present study, we developed transient ischemia in the forebrain of the gerbil and found cognitive impairment using the Barnes maze test and passive avoidance test for spatial memory and learning memory, respectively. In addition, neuronal loss/death was detected in the Cornu Ammonis 1 (CA1) region of the gerbil hippocampus after the ischemia by cresyl violet histochemistry, immunohistochemistry for neuronal nuclei and histofluorescence with Fluoro-Jade B. Furthermore, in the CA1 region following ischemia, myelin and vesicular synaptic density were significantly decreased using immunohistochemistry for myelin basic protein and vesicular glutamate transporter 1. In the gerbils, treatment with COG-up^®^ (a combined extract of *Erigeron annuus* (L.) Pers. and *Brassica oleracea* Var.), which was rich in scutellarin and sinapic acid, after the ischemia, significantly improved ischemia-induced decline in memory function when compared with that shown in gerbils treated with vehicle after the ischemia. In the CA1 region of these gerbils, COG-up^®^ treatment significantly promoted the remyelination visualized using immunohistochemistry myelin basic protein, increased oligodendrocytes visualized using a receptor-interacting protein, and restored the density of glutamatergic synapses visualized using double immunofluorescence for vesicular glutamate transporter 1 and microtubule-associated protein, although COG-up^®^ treatment did not protect pyramidal cells (principal neurons) located in the CA1 region form the ischemic insult. Considering the current findings, a gerbil model of ischemic stroke apparently showed cognitive impairment accompanied by ischemic injury in the hippocampus; also, COG-up^®^ can be employed for improving cognitive decline following ischemia-reperfusion injury in brains.

## 1. Introduction

Ischemia/reperfusion injury in the brain is a common property of ischemic stroke, which involves a period of impaired blood circulation in the brain, followed by restoration of perfusion through medical intervention [1,2]. It was acknowledged that cognitive impairment is developed by brain ischemia/reperfusion injury which involves various neurological and behavioral changes such as paralysis in limbs, vertigo, confusion, cognitive dysfunction [3,4,5].

It is well accepted that the hippocampus plays a pivotal role in memory and cognitive function [6,7]. Therefore, ischemia/reperfusion in the hippocampus induces neuronal damage or death, which is accompanied by decreases in synaptic density, demyelination and axonal damage which can lead to memory and cognitive deficits [3,8,9,10]. It is well established that, in the hippocampus of the gerbil, delayed neuronal death is induced in the CA 1 region four to five days after in five-minute transient ischemia (TI) in the forebrain [8,11,12]. For this property, many studies have utilized this model to develop cognitive impairment followed by delayed neuronal death [3,8,13].

Many precedent studies show that the enhancement of remyelination after ischemic insults is important for the functional recovery of memory and cognition [3,8,14]. In particular, oligodendrocytes, a type of glial cells, form myelin sheath enveloping axons in the brain and spinal cord in order to accelerate neural transmission by saltatory conduction, and newly generated oligodendrocytes play an important role in remyelination [15,16,17]. In addition, accumulated clinical and preclinical data demonstrate that change in glutamate neurotransmission in the brain may be linked to cognitive impairment [18,19].

Numerous studies have reported the beneficial effects of medicinal herbs. *Erigeron annuus* (L.) Pers. (EALP) belongs to the Asteraceae family, and its extract shows anti-inflammatory effects in lipopolysaccharide-induced activated macrophage and carrageen-induced acute inflammation in rat paws [20]. In addition, the genus *Brassica* belonging to the Brassicaceae family has beneficial attributes. For instance, the extract of *Brassica oleracea* Var. *Italica* attenuates amyloid β_1–42_-induced learning and memory impairment in mice [21]. However, to the best of our knowledge, experiments on the effects of the extracts from *Erigeron annuus* (L.) Pers. and *Brassica oleracea* Var. on the improvement of cognitive impairment following TI in rodent brains have rarely been conducted. Therefore, herewith we developed transient ischemia in the forebrain using gerbils, analyzed COG-up^®^ (a combined extract of *Erigeron annuus* (L.) Pers. and *Brassica oleracea* Var.) and investigated whether COG-up^®^ improve cognitive impairment induced by TI in the hippocampus. In addition, we examined the effects of COG-up^®^ on remyelination and restoration of synaptic density in the damaged hippocampus.

## 2. Materials and Methods

### 2.1. Experimental Animals

Eighty-four male gerbils at the age of six months (85 ± 5 g of body weight) were provided by Experimental Animal Center of Kangwon National University (Chuncheon, Gangwon, Korea). The gerbils were housed in conventional room with optimum conditions (24 ± 1 °C of room temperature; 50 ± 5% of relative humidity). A steady cycle of light and dark was controlled every 12 h, and pellet feed (DBL Co. Ltd., Chungbuk, Korea) and water were freely accessible.

All experimental processes were according to the guidelines described in the “Current International Laws and Policies”, a part of the “Guide for the Care and Use of Laboratory Animals”. Approval for the experimental protocol was sanctioned by Institutional Animal Care and Use Committee of Kangwon National University (Chuncheon, Korea) on 18 February 2020 (approval no., KW-200113-1).

### 2.2. Experimental Groups

In this study, four groups were used: (1) sham+vehicle group (*n* = 21) which was given sham surgery and treated with vehicle (saline); (2) TI+vehicle group (*n* = 21) which was given TI surgery and treated with vehicle; (3) sham+COG-up^®^ group (*n* = 21) which was undergone sham operation and treated with 100 mg/kg COG-up^®^; and (4) TI+COG-up^®^ group (*n* = 21) which was undergone TI operation and treated with 100 mg/kg COG-up^®^.

### 2.3. Qualitative Analysis of COG-Up^®^

COG-up^®^ (a combined extract of *Erigeron annuus* (L.) Pers. and *Brassica oleracea* Var.) was provided by Famenity (Uiwang, Korea). Scutellarin (Glentham, Corsham, UK) standard sample and test sample (COG-up^®^) were precisely weighed and dissolved. These samples (5 μL, respectively) were subjected to HPLC (Agilent 1260 Infinity II Prime LC System) (Agilent Technologies, Inc., Waldbronn, Germany) using Discovery C18 column (diameter of 4.6 mm, length of 250 mm) (Sigma-Aldrich Co., St. Louis, MO, USA), which was filled with octadecylsilyl silica gel (diameter of 5 μm) at 1.0 mL/min of flow rate. Optimum HPLC separation was achieved at 30 °C. UV wavelength was 335 nm for the scutellarin. Phosphoric acid (Junsei Chemical Co., Ltd., Tokyo, Japan) was mixed with distilled water and methanol (JT Baker, Phillipsburg, NJ, USA) which were used as a mobile phase. Mobile phase condition for the scutellarin using A (0.5% phosphoric acid in water) and B (methanol) was as follows: 0–5 min (25% B), 5–25 min (25–55% B), 25–30 min (55–100% B), 30–32 min (100% B), 32–33 min (100–25% B), 33–40 min (25% B).

For high analytical performance, pretreatment using alkaline solvent was required for sinapic acid. Sinapic acid (Sigma-Aldrich Co., St. Louis, MO, USA) standard sample and the test sample (COG-up^®^) were precisely weighed and dissolved. These samples (5 μL) were subjected to HPLC (Agilent 1260 infinity II, Agilent Technologies, Inc., Santa Clara, CA, USA) using the same column and flow rate. Optimum HPLC separation was achieved at 30 °C. UV wavelength was 320 nm for sinapic acid. The mobile phase condition for sinapic acid using the same solution A and B was as follows: 0–15 min (10–30% B), 15–25 min (30% B), 25–26 min (30–50% B), 26–27 min (50–90% B), 27–30 min (90% B), 30–32 min (90–10% B) and 32–37 min (10% B).

### 2.4. Induction of TI and Treatment with COG-Up^®^

The gerbils underwent TI in the forebrain to develop cognitive impairment due to ischemic damage [3]. As described previously [22], the animals were adequately anesthetized with 2.5% isoflurane (Hana Pharmaceutical Co. Ltd., Seoul, Korea) (in mixture of 67% N_2_O and 33% O_2_) using an inhaler. Under the anesthesia, an incision was made on the midline of the ventral neck. Bilateral common carotid arteries (BCCA) were freed from the vagus nerve and occluded with aneurysm clips (0.69 N) (Yasargil FE 723K; Aesculap, Tuttlingen, Germany) for five minutes. For the complete occlusion of the BCCA, the stop of blood flow was verified through observing in right and left central arteries (branches of the internal carotid arteries) located at both retinae using ophthalmoscope (HEINE K180^®^) (Heine Optotechnik; Herrsching, Germany). The aneurysm clips were removed at five minutes after ligating the BCCA, and the skin was sutured with 3–0 suture silk (Ethicon Inc, Somerville, NJ, USA). In this study, sham TI operation was performed like the identical surgical procedure, excepting BCCA ligation. In particular, body temperature was controlled at normothermia (37 ± 0.2 °C) during the surgical procedure using rectal temperature probe (TR-100) (Fine Science Tools Inc.; Foster City, CA, USA).

In this study, vehicle and COG-up^®^ were orally administrated once a day five days to 30 days after TI.

### 2.5. Barnes Maze Test (BMT)

**Figure 1:**
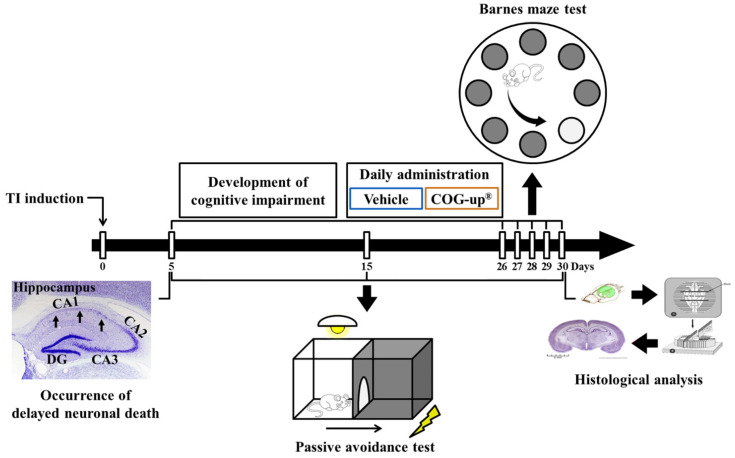
Experimental timeline. Cognitive impairment is developed for 25 days from five days after TI operation. Gerbils are daily treated with vehicle or COG-up^®^ from five days to 30 days after TI. Delayed neuronal death is examined five days after TI. Passive avoidance test is conducted zero, five, 15 and 30 days after TI, and Barnes maze test is carried out from 26 days to 30 days after TI. Histological examination is performed five, 15 and 30 days after TI.

To examine spatial memory, BMT was daily conducted 26 to 30 days after TI (Figure 1). According to a published method [3], visual signs were located around the maze at a level that would be perceptible to the gerbils. Lights and a stereo speaker were installed beneath the ceiling in order to maintain brightness (220 lx) and background sound (85 dB). The gerbils were adapted to a refuge for two minutes on the first day of training. Each gerbil was given training three times per day with 15 min of intervals per day for four successive days. When the gerbil failed to find the refuge within 180 s, we escorted the gerbil toward the escape hole and let the gerbil stay for 35 s in the refuge. Thereafter, the gerbil was placed onto the center of the maze to explore an escape hole that is linked to the refuge. Each trial was finished when the gerbil had entered the refuge and stayed for 30 s. The substantial test was carried out one day after the final training. The refuge was removed, and, when the gerbil went to the entry area where the refuge had been previously located, the latency time was recorded within 90 s.

### 2.6. Passive Avoidance Test (PAT)

In accordance with some references [3,8], PAT was performed to investigate learning memory on day 5, day 15 and day 30 after TI (Figure 1). We used Gemini Avoidance System (GEM 392) (San Diego Instruments Inc., San Diego, CA, USA) for PAT. This apparatus consisted of a dark and light compartment that was connected through a vertical gate between the compartments. The experimental procedure was separated into two sections: training section and substantial trial section. The training was performed one day before each substantial trial. For the training, the gerbil was allowed to freely explore the dark and light compartment for 60 s. Thereafter, the vertical door was shut as soon as the gerbil entered the dark compartment, and the animals received an inescapable foot-shock of 0.5 mA for five seconds. For the substantial trial, each animal was put in the light compartment, and the latency time to go into the dark compartment was evaluated within 180 s.

### 2.7. Preparation of Brain Sections

The brain tissue sections containing the hippocampus were prepared to carry out histological analyses in the four groups five days (*n* = 7 in each group), 15 days (*n* = 7 in each group) and 30 days (*n* = 7 in each group) after TI. According to previously published methods [3,23], the gerbils of the four groups (*n* = 21, respectively) were profoundly anesthetized with 200 mg/kg pentobarbital sodium (intraperitoneal injection) (JW Pharmaceutical Co., Ltd., Seoul, South Korea) [22]. Under anesthesia, the gerbils were perfused (flow rate of 6 mL/min, total perfused volume of 60 mL) with 100 mM phosphate-buffered saline (PBS, pH 7.4) and subsequently fixed with paraformaldehyde solution (4% *w/v*; in 100 mM phosphate buffer (PB), pH 7.4) with same flow rate and total perfused volume via the ascending aorta. When their brains were fixed, the brains were harvested and individually immersed in the same fixative for post-fixation for six hours at room temperature. Thereafter, these brains were infiltrated with 30% *w/v* sucrose (in 100 mM PB) to be cryoprotected for 24 h at room temperature. The brain tissues were serially cut into 30-μm coronal sections using sliding microtome (SM2020 R) (Leica, Nussloch, Germany) equipped with freezing stage (BFS-40MP) (Physitemp Instruments Inc., Clifton, NJ, USA), Representative sections were selected at antero-posterior of −1.8 mm to 2.7 mm from the bregma with a reference of the “Brain Atlas of the Mongolian Gerbil (*Meriones unguiculatus*)” [24].

### 2.8. Cresyl Violet (CV) Staining

CV staining was carried out to examine the change in the distribution of hippocampal cells. As described in a previous paper [25], the brain sections were mounted onto the slide glasses coated with gelatin. After confirming their attachment, the prepared brain sections were immersed in 0.1% *w/v* CV acetate (Sigma-Aldrich Co., St. Louis, MO, USA) for 20 min. Thereafter these stained sections were briefly washed followed by decolorized in 50% ethanol for two minutes, subsequently dehydrated through successive incubation in 70%, 80%, 90%, 95% and 100% ethanol and then cleared in xylene. These stained sections were mounted with cover glasses.

To observe the changes of the CV-stained cells in gerbil hippocampus, images of the cells stained with CV were taken using microscope (BX53) (Olympus, Tokyo, Japan).

### 2.9. Fluoro-Jade B (FJB) Histofluorescence

FJB histofluorescence was performed to examine neuronal death (loss) in the hippocampus with reference to some previous reports [26,27]. The brains sections were put onto the slide glasses, which were coated with gelatin. These sections were soaked in 0.06% KMnO_4_ (Sigma-Aldrich Co., St. Louis, MO, USA) for 20 min and briefly washed. After washing, these sections were soaked in 0.0004% FJB (Histo-chem Inc., Jefferson, AR, USA) for 30 min and rinsed. Thereafter, these sections were placed onto slide warmer until they were completely dried. The reacted sections were finally cleared in xylene and coverslipped with dibutyl phthalate polystyrene xylene (Sigma-Aldrich Co., St. Louis, MO, USA).

In order to count numbers of FJB positive cells, five sections per gerbil were chosen. According to a paper [28] with some modification, the stained sections were observed using epifluorescent microscope (Carl Zeiss, Göttingen, Germany) with blue excitation fluorescent filter (wavelength of 450–490 nm). Images of FJ B positive cells, which underwent degeneration (bright fluoresce) when compared with the background [26], were captured and counted in 250 × 250 μm at the middle of the CA1 region. Finally, the mean number of FJB positive cells was calculated using NIH Image 1.59 software (NIH, Bethesda, Rockville, MD, USA).

### 2.10. Immunohistochemistry

To examine changes in neurons, myelin and oligodendrocytes in the hippocampus, immunohistochemistry was carried out using avidin–biotin complex (ABC) method. According to previous studies [25,29] with minor modifications, the prepared sections were washed with PBS (pH 7.4) and soaked in 0.3% H_2_O_2_ (in 100 mM PBS, pH 7.4) for 30 min to block endogenous peroxidase activity. Subsequently, to block non-specific immunoreaction, these sections were incubated in five percent goat serum or horse (in 100 mM PBS, pH 7.4) for 30 min. Next, the sections were immunoreacted with primary antibodies (Table 1) for 24 h at 4 °C. Thereafter, the immunoreacted sections were incubated in each secondary antibody (Table 1) for two hours at room temperature followed by ABC (diluted 1:300) (Vector Laboratories, Burlingame, CA, USA). After briefly washing, these sections were reacted with 0.06% 3, 3′-diaminobenzidine tetrahydrochloride (Sigma-Aldrich Co, St Louis, MO, USA) (in 100 mM PBS containing 0.1% H_2_O), washed with 100 mM PBS (pH 7.4), mounted onto the microscopic slides, dehydrated in 70%, 80%, 90%, 95% and 100% ethanol and cleared in xylene. Lastly, these stained sections were coverslipped with Canada balsam (Kanto Chemical Co., Inc., Tokyo, Japan).

For negative controls, adjacent tissue sections were immersed in pre-immune serum without each primary antibody. After testing, immunoreactive structures were not shown in the sections (data not shown).

In order to evaluate changes in neurons and oligodendrocytes, the numbers of NeuN immunoreactive neurons and RIP immunoreactive oligodendrocytes, five sections/gerbil were selected and analyzed using microscope (BX53) (Olympus, Tokyo, Japan) in the same way described above (in Section 2.8).

To evaluate changes in myelin, the optical density of MBP immunoreactive structure was presented. In accordance with a previous study [30], five sections/gerbils were chosen, and the image of MBP immunoreactive structure was taken using microscope (BX53) equipped with cellSens Standard software (Olympus, Tokyo, Japan). The captured image was converted to eight bits of grey scale (range, 0–255; from black to white) to measure grey scale intensity. Average density of MBP immunoreactive structure was computed using Image J software (version 1.46) (National Institutes of Health, Bethesda, Rockville, MD, USA). Lastly, the density of the MBP immunoreactive structure was presented as relative optical density (ROD) as percentage of the Sham+vehicle group (100%).

### 2.11. Double Immunofluorescence

Double immunofluorescence was performed to examine synaptic formation by examining the co-localization of vesicular glutamate transporter 1 (VGLUT-1; a marker for excitatory synapse) and microtubule-associated protein 2 (MAP2; a marker for apical dendrites) immunoreactive structures. As described previously [3], in brief, rabbit anti-VGLUT-1 (diluted 1:500) (Synaptic Systems GmbH, Göttingen, Germany) and mouse anti-MAP2 (diluted 1:400) (Chemicon International Inc., Temecula, CA, USA) were used as primary antibodies. The sections were reacted with secondary antibody—mixture of Alexa Fluor^®^ 488-conjugated donkey anti-mouse IgG (diluted 1:500) (Invitrogen, Waltham, MA, USA) and Alexa Fluor^®^ 546-conjugated goat anti-rabbit IgG (diluted 1:500) (Invitrogen, Waltham, MA, USA).

In accordance with our previous study [3], in each group, five sections at each time were observed using epifluorescent microscope (Carl Zeiss, Göttingen, Germany) with blue (450–490 nm of wavelength; for observing VGLUT-1 immunoreactive structure) and green (510–560 nm of wavelength; for observing MAP2 immunoreactive structure) excitation fluorescent filters. Using cellSens Standard software (Olympus, Tokyo, Japan), each VGLUT-1 and MAP2 immunoreactive structure was captured and merged. To quantitatively analyze the co-localized VGLUT-1/MAP2 immunoreactive structure, the merged image was converted into grey scale with 0–255 of range from black and white. Using Image J software (version 1.46) (National Institutes of Health, Bethesda, Rockville, MD, USA), the density of the co-localized VGLUT-1/MAP2 immunoreactive structure was presented as ROD as %: the ROD of the sham+vehicle group was designated as 100%.

### 2.12. Statistical Analysis

To perform all statistical analyses, in this study, SPSS software (version 15.0) (SPSS Inc., Chicago, IL, USA) was used. In addition, Kolmogorov and Smirnov test to evaluate normal distributions and Bartlett test to evaluate identical standard error of the mean (SEM) were used. Moreover, all presented data were taken for the normality test. The statistical significances of the mean among the experimental groups were determined by two-way analysis of variance followed by post hoc Tukey’s test for all pairwise multiple comparisons. All presented data were shown as the mean ± SEM, and statistical significance was designated when *p*-value was less than 0.05.

## 3. Results

### 3.1. Major Ingredients of COG-Up^®^

**Figure 2:**
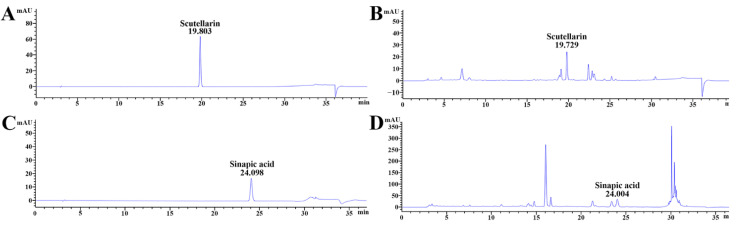
Qualitative analysis of COG-up^®^ via high-performance liquid chromatography (**A**,**B**) are produced under the same mobile phase condition for the analysis of scutellarin. (**C**,**D**) were produced under the same mobile phase condition for the analysis of sinapic acid. The retention time of the standard scutellarin and sinapic acid was 19.803 and 24.098 min, respectively. The retention time of scutellarin and sinapic acid in COG- up^®^ was 19.729 and 24.004 min, respectively.

Scutellarin standard was 19.803 min at the retention time, as shown in Figure 2A, which was also detected in COG-up^®^ (19.729 min of retention time (Figure 2B). The retention time of sinapic acid standard was 24.098 min (Figure 2C), which was also shown in COG-up^®^ (24.004 min of retention time) (Figure 2D). Accordingly, scutellarin and sinapic acid were revealed as major ingredients of the COG-up^®^.

### 3.2. Cognitive Function

#### 3.2.1. Spatial Memory by BMT

**Figure 3:**
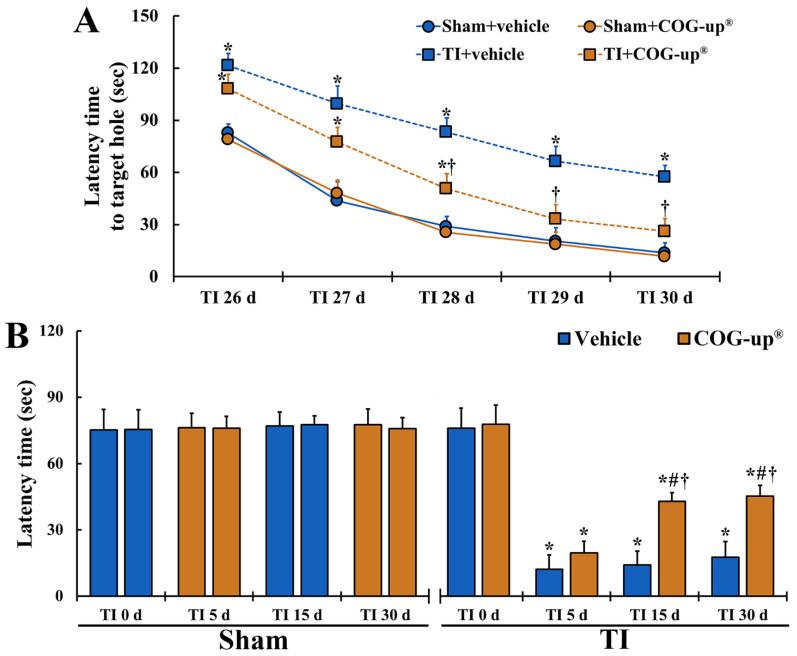
(**A**) Spatial memory by BMT. In the TI+COG-up^®^ group, latency time in the BMT is significantly shortened from 28 days after TI as compared with the TI+vehicle group. The bars indicate mean ± SEM (*n* = 7 at each time in each group; * *p* < 0.05 versus sham+vehicle group and ^†^ *p* < 0.05 versus corresponding time of TI+vehicle group). (**B**) Learning memory by PAT. In the TI+COG-up^®^ group, latency time 15 and 30 days after TI is significantly increased as compared with the TI+vehicle group. The bars indicate mean ± SEM (*n* = 7 at each time in each group; * *p* < 0.05 versus sham+vehicle group, ^#^ *p* < 0.05 versus prior time of each group, and ^†^ *p* < 0.05 versus corresponding time of TI+vehicle group).

As shown in Figure 3A, in all four groups, the latency time to find the escape hole evaluated from 26 days to 30 days after TI was gradually shortened. Latency time in the two sham groups was not different from each other and latency time in the TI+vehicle group was significantly longer than that shown in the sham groups. In the TI+COG-up^®^ group, latency time was also significantly longer than that shown in the sham groups 26, 27 and 28 days after TI. However, in this group, latency time on day 28, day 29 and day 30 after TI was significantly shortened when as compared to that shown in the TI+vehicle group.

#### 3.2.2. Learning Memory by PAT

In all four groups, latency time evaluated at zero-day after TI did not exhibit differences (Figure 3B). In both sham groups, latency time shown at every time point after sham TI operation was similar to that found at zero-day (Figure 3B). After TI, latency time in the TI+vehicle group was significantly short as compared with that shown in the sham groups, although the latency time was gradually increased with time after TI (Figure 3B). In the TI+COG-up^®^ group, latency time shown on day 5 after TI was increased as compared to the TI+vehicle group, but significant difference in the latency time was not found between the two groups (Figure 3B). However, latency time at 15 and 30 days after TI was significantly longer as compared to that found in the TI+vehicle group (Figure 3B).

### 3.3. Cellular Change in the Hippocampus

**Figure 4:**
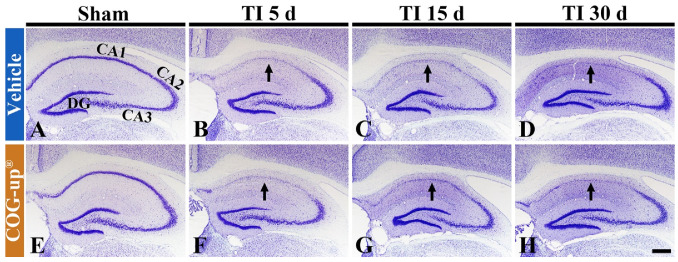
CV staining in the hippocampus of the sham+vehicle (**A**), sham+COG-up^®^ (**E**), TI+vehicle and TI+COG-up^®^ groups five (**B**,**F**), 15 (**C**,**G**) and 30 (**D**,**H**) days after TI. In the TI+vehicle and TI+COG-up^®^ groups, CV stainability is decreased in the stratum pyramidale (arrows) of the CA1 region from five days after TI: the distribution pattern of CV-stained cells is not changed until 30 days after TI. Scale bar = 400 μm. DG, dentate gyrus.

To examine change in cells in the gerbil hippocampus following TI, CV staining was performed. Cells stained by CV, in all sham groups, were easily identified in the hippocampus (Figure 4A,E). In particular, CV-stained cells formed the stratum pyramidale (SP): these cells are principal cells and called pyramidal cells (Figure 4A,E). Five days after TI, in the TI+vehicle and TI+COG-up^®^ groups, the CV stainability was decreased in the SP of the CA1 region, not CA2 and 3 regions (Figure 4B,F). This finding means that delayed neuronal damage/death occurs only in the CA1 region following TI. Thereafter, in these two groups, the distribution pattern of CV-stained cells was not altered until 30 days after TI (Figure 4C,D,G,H).

### 3.4. TI-Induced Neuronal Death (Loss) in the CA1 Region

#### 3.4.1. Findings by NeuN Immunohistochemistry

**Figure 5:**
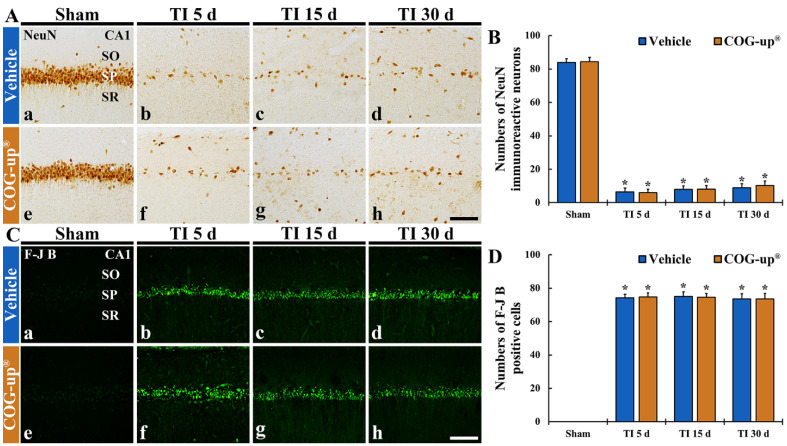
(**A**,**B**) Immunohistochemistry for NeuN (**A**) and FJB histofluorescence (**C**) in the CA1 region of the sham+vehicle (**Aa**,**Ca**), sham+COG-up^®^ (**Ae**,**Ce**), TI+vehicle and TI+COG-up^®^ groups five (**Ab**,**Af**,**Cb**,**Cf**), 15 (**Ac**,**Ag**,**Cc**,**Cg**) and 30 (**Ad**,**Ah**,**Cd**,**Ch**) days after TI. In both TI+vehicle and TI+COG-up^®^ groups, NeuN immunoreactive cells are rarely detected, and numerous FJB positive cells are shown. Scale bar = 100 μm. (**B**,**D**) Mean numbers of NeuN immunoreactive cells (**B**) and mean numbers of FJB positive cells (**D**). SO, stratum oriens; SP, stratum pyramidale; SR, stratum radiatum. The bars indicate mean ± SEM (*n* = 7, respectively; * *p* < 0.05 versus sham+vehicle group).

In all sham groups, numerous NeuN immunoreactive pyramidal cells stained with NeuN were located in the SP of the CA1 region (about 84 cells/250 μm^2^) (Figure 5(Aa,Ae)). In the TI+vehicle and TI-COG-up^®^ groups, NeuN immunoreactive cells were rarely detected in the SP five days after TI, showing that their number was about nine cells/250 μm^2^ (Figure 5(Ab,Af,B)). In these two groups, at 15 and 30 days after TI, the number of NeuN immunoreactive pyramidal cells was not significantly different from that shown five days after TI (Figure 5(Ac,Ad,Ag,Ah,B)). This finding means that treatment with COG-up^®^ does not influence TI-induced delayed neuronal death.

#### 3.4.2. Findings by FJB Histofluorescence

In both sham+vehicle and sham+COG-up^®^ groups, FJB positive cells were not detected in the CA1 region (Figure 5(Ca,Ce)). In the TI+vehicle and TI+COG-up^®^ groups, a plenty number of FJB positive cells (about 74 cells/250 μm^2^) were observed in the SP five days after TI (Figure 5(Cb,Cf,D)). In these groups, the numbers of FJB positive cells found 15 and 30 days after TI were not different from those found five days after TI (Figure 5(Cc,Cd,Cg,Ch,D)). Definitely, this finding shows that treatment with COG-up^®^ does save CA1 pyramidal cells from TI-induced death.

### 3.5. Myelin Using MBP Immunohistochemistry

**Figure 6:**
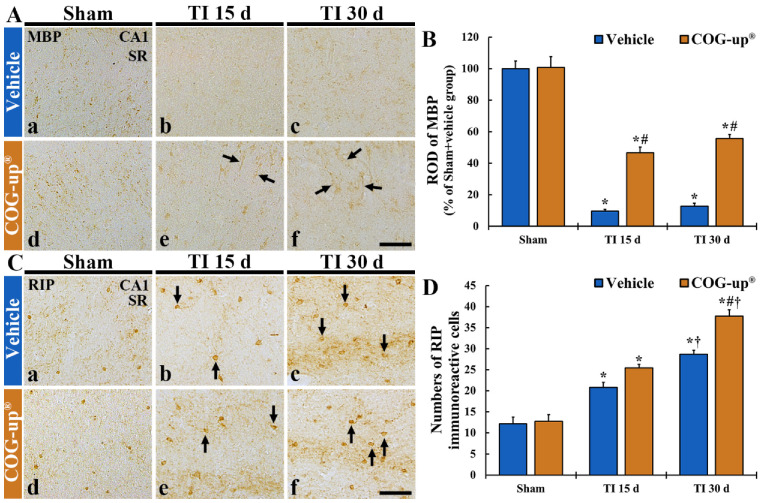
(**A**,**B**) Immunohistochemistry for MBP (**A**) and RIP (**C**) in the CA1 region of the sham+vehicle (**Aa**,**Ca**), sham+COG-up^®^ (**Ae**,**Ce**), TI+vehicle and TI+COG-up^®^ groups at five (**Ab**,**Af**,**Cb**,**Cf**), 15 (**Ac**,**Cc**) and 30 (**Ad**,**Cd**) days after TI. In the TI+vehicle group, MBP immunoreactive nerve fibers are decreased at 15 and 30 days after TI. However, in the TI+COG-up^®^ group, MBP immunoreactive structures (arrows) are significantly increased at 15 and 30 days after TI when compared to those shown in the TI+vehicle group. RIP immunoreactive cells (arrows) are increased in both TI+vehicle and TI+COG-up^®^ group, at 15 and 30 days after TI; however, RIP immunoreactive oligodendrocytes in the TI+ COG-up^®^ group are more than those shown in the TI+vehicle group. SO, stratum oriens; SP, stratum pyramidale; SR, stratum radiatum. Scale bar = 100 μm. (**B**,**D**) ROD of MBP immunoreactive structure (**B**) and mean numbers of RIP immunoreactive oligodendrocytes (**D**). The bars indicate mean ± SEM (*n* = 7 at each time in each group; * *p* < 0.05 versus sham+vehicle group, ^#^ *p* < 0.05 versus prior time point of each group, and ^†^ *p* < 0.05 versus corresponding time of TI+vehicle group).

In all sham groups, MBP immunoreactive structures, as myelinated nerve fibers, were distributed throughout all layers in the CA1 region (Figure 6(Aa,Ad)). In the TI+vehicle group, the density of MBP immunoreactive structures was significantly decreased (about 9% and 12% on day 15 and day 30 after TI, respectively, versus sham+vehicle group) as compared with that shown in the sham+vehicle group (Figure 6(Ab,6Ac,B)). In contrast, in the TI+COG-up^®^ group, the density of MBP immunoreactive structure was significantly higher (about 46% and 55% at 15 and 30 days, respectively, after TI versus sham+vehicle group) than that evaluated in the TI+vehicle group (Figure 6(Ae,Af,B)).

### 3.6. Oligodendrocytes Using RIP Immunohistochemistry

In both sham+vehicle and sham+COG-up^®^ groups, RIP immunoreactive structures, as oligodendrocytes, were clearly shown in all layers of the CA1 region (Figure 6(Ca,Cd)). In the TI+vehicle group, the number of RIP immunoreactive oligodendrocytes was significantly increased (about 20 cells/250 μm^2^ and 28 cells/250 μm^2^ at 15 and 30 days, respectively, after TI) as compared to that evaluated in the sham+vehicle group (Figure 6(Cb,Cc,D)). In the TI+COG-up^®^ group, however, a significantly increased number of RIP immunoreactive oligodendrocytes was detected (about 25 cells/250 μm^2^ and 37 cells/250 μm^2^ on day 15 and day 30 after TI, respectively) when compared with that evaluated in the TI+vehicle group (Figure 6(Ce,Cf,D)).

### 3.7. Synaptic Density Using Double Immunofluorescence for VGLUT-1/MAP2

**Figure 7:**
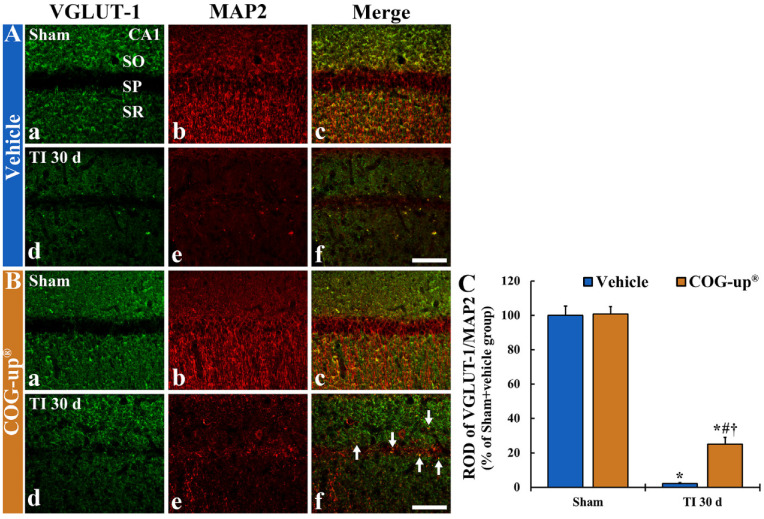
(**A**,**B**) Double immunofluorescence for VGLUT-1 (**a**,**d**; green)/MAP2 (**b**,**e**; red), and merged images (**c**,**f**) in the CA1 region of the sham+vehicle (**Aa**–**Ac**), sham+COG-up^®^ (**Ba**–**Bc**), TI+vehicle (**Ad**–**Af**) and TI+COG-up^®^ (**Bd**–**Bf**) groups at 30 days after TI. In the TI+vehicle group, VGLUT-1/MAP2 immunoreactive structures are hardly found. However, in the TI+COG-up^®^ group, co-localized VGLUT-1/MAP2 immunoreactive structures are easily shown (arrows). SO, stratum oriens; SP, stratum pyramidale; SR, stratum radiatum. Scale bar = 100 μm. (**C**) ROD of VGLUT-1/MAP2 fluorescent structure. The bars indicate mean ± SEM (*n* = 7 at each time in each group; * *p* < 0.05 versus sham+vehicle group, ^#^ *p* < 0.05 versus prior time point of each group, and ^†^ *p* < 0.05 versus corresponding time of TI+vehicle group).

In all sham groups, VGLUT-1 immunoreactive structures, as glutamate transporter in the membrane of synaptic vesicles, were predominately distributed in the stratum oriens and stratum radiatum (Figure 7(Aa,Ba)). The VGLUT-1 immunoreactive structures were co-localized with MAP2 immunoreactive structures, as neuronal dendritic extensions (main dendrites), in the CA1 region (Figure 7(Ab,Ac,Bb,Bc)). In the TI+vehicle group, when VGLUT-1/MAP2 immunoreactive structures were observed at 30 days after TI, they were significantly decreased (about 2% versus sham+vehicle group) as compared to the sham+vehicle group (Figure 7(Ad–Af),C). However, in the TI+COG-up^®^ group at 30 days after TI, a significant increase in VGLUT-1/MAP2 immunoreactive structures were found (about 25% versus TI+vehicle group) when compared with the TI+vehicle group (Figure 7(Bd–Bf),C).

## 4. Discussion

In the present study, cognitive impairment was apparently induced in a gerbil model of TI which was accompanied by the death of pyramidal cells (neurons as principal cells) of the hippocampal CA1 region as shown by CV staining, NeuN immunohistochemistry and FJB histofluorescence. In addition, in the CA1 region following ischemia, myelin and vesicular synaptic density were significantly decreased as shown by immunohistochemistry for MBP and VGLUT-1. Therefore, we had tried to investigate whether COG-up^®^ treatment improved microenvironmental damages such as demyelination (loss of myelin) and decreased synaptic density in the hippocampus.

Accumulating experimental data show that herbal medicines were used thanks to their beneficial attributes such as anti-inflammatory and antioxidant activities [12,20,31,32]. Furthermore, many studies have investigated active ingredients of herbal medicines. For instance, the therapeutic effect of *Angelica gigas* Nakai (Umbelliferae family) root extract containing decursin (a coumarin derivate compound) was shown in the ischemic hippocampus via protecting blood–brain barrier leakage and astrocyte endfeet damage in a gerbil model of TI [33]. Additionally, it was reported that, in a gerbil model of TI, pretreated with YES-10^®^, a combined extract of EALP and *Clematis mandshurica* RUPR. (Ranunculaceae family), containing scutellarin and chlorogenic acid showed strong neuroprotective effect in the hippocampus after TI [12]. We, in this study, investigated the major ingredients of COG-up^®^; and, as shown in the HPLC result, we found scutellarin and sinapic acid as active ingredients of COG-up^®^. A precedent study reported that scutellarin, as a flavonoid glycoside compound, ameliorated learning and memory deficit induced by chronic cerebral hypoperfusion in rats [34]. In addition, Kim et al. (2011) reported that synaptic acid treatment attenuated memory impairment in a rat model of global cerebral ischemia induced by ligation of four vessels [35].

Recently, studies developed combined extracts originating natural resources and reported that they improved cognitive dysfunctions following dementia. For example, Shenmayizhi decoction, a Chinese herbal prescription, consists of four herbal extracts—*Panax ginseng* C. A. Mey. (Araliaceae family), *Gastrodia elata* Bl. (Orchidaceae family), *Ligusticum chuanxiong* Hort. (Apiaceae family) and *Euonymus alatus* Sieb. (Celastraceae family)—ameliorate declined memory and learning function in a rat model of vascular cognitive impairment induced by BCCA occlusion [36]. Based on these previous studies, in this experiment, we treated COG-up^®^ after TI in gerbils and found that TI-induced cognitive impairment was significantly improved when we performed BMT and PAT for spatial memory and learning memory, respectively.

In the present study, although COG-up^®^ treatment after TI showed an improvement of cognitive impairment in the gerbils, the COG-up^®^ treatment failed to protect CA1 pyramidal cells from TI when we performed CV histochemistry, NeuN immunohistochemistry and FJB histofluorescence. Therefore, we had tried to investigate whether COG-up^®^ treatment improved microenvironmental damages such as demyelination (loss of myelin) and decreased synaptic density in the hippocampus.

As reviewed by Baaklini et al. (2019), demyelination attributed to various pathological conditions retards axonal conduction and may bring a failure to transmit neural information, passing by demyelinated segments [37]. Demyelinated axons due to injuries undergo remyelination for the functional recovery of neural activities, and this process is facilitated by newly generated oligodendrocytes [37,38]. It was demonstrated that the improvement of cognitive impairment induced by ischemic stroke is accompanied by remyelination and proliferation of oligodendrocytes. For example, Chen et al. (2018) reported that treatment with *N*-acetyl-5-methoxytryptamine (melatonin), a lipophilic hormone synthesized by the pineal gland, after ischemic stroke in gerbils, excellently improved memory impairment and showed remyelination in the hippocampus [3]. Furthermore, Qu et al. (2014) demonstrated that quercetin, a flavonoid abundant in diverse plants, improved cognitive deficit induced by hypoxia-ischemia via promoting remyelination in neonatal rats [14]. With correspondence to these precedent data, in this study, COG-up^®^ treatment after TI in the gerbils increased MBP immunoreactive structure (myelin) and RIP immunoreactive cells (oligodendrocytes) in the ischemic CA1 region.

In our current study, COG-up^®^ treatment after TI in the gerbils increased the density of glutamatergic synapses in the ischemic CA1 region using double immunofluorescence for VGLUT-1/MAP2. Recently, we reported that treatment with melatonin after TI in gerbils improved the expression level of VGLUT-1 (higher synaptic density) when compared with that evaluated in ischemic gerbils treated with vehicle [3]. Glutamate, as a major excitatory neurotransmitter in the brain, contributes to aspects of higher intellectual function [39]. It is acknowledged that glutamatergic neurotransmission is mainly achieved by VGLUT-1 [40]. A study described that VGLUT-1-knockout mice displayed a decline in hippocampal long-term potentiation which was in company with impairment of spatial memory [41]. In addition, Cao et al. (2016) showed that the expression level of VGLUT-1 was decreased in the hippocampal CA1–3 regions accompanied with cognitive impairment after chronic cerebral ischemia induced by BCCA occlusion in rats [42]. Moreover, a case-control autopsy study by Kirvell et al. (2010) showed that, in patients with stroke, the preservation of glutamatergic synapses in the frontal cortex against the temporal cortex might play a role in maintaining cognitive function against dementia following a stroke [39]. 

## 5. Conclusions

Our present data showed that COG-up^®^ contained scutellarin and sinapic acid as major ingredients. Treatment with COG-up^®^ after TI in gerbils improved cognitive impairment (decline in spatial and learning memory) induced by TI. However, COG-up^®^ treatment did not protect against the death of pyramidal cells (principal cells) located in the hippocampal CA1 region following TI. Instead, we found that treatment with COG-up^®^ improved remyelination and restored the density of glutamatergic synapses in the ischemic CA1 region. Based on this result, we suggest that follow-up studies such as mechanisms and optimization improving cognitive function need to be conducted; through further studies, COG-up^®^ can be employed for improving cognitive decline following ischemic stroke via commercializing as health/functional foods and medicines. Ultimately, it can enhance national health.

## Figures and Tables

**Table 1:** Primary and secondary antibodies for immunohistochemical staining.

**Primary Antibodies**	**Dilution**	**Suppliers**
Mouse anti-neuronal nuclei (NeuN)	1:1000	Chemicon, Temecula, CA, USA
Rabbit anti-myelin basic protein (MBP)	1:200	Abcam, Cambridge, UK
Mouse anti-receptor interacting protein (RIP)	1:200	Santa Cruz Biotechnology, Santa Cruz, CA, USA
**Secondary Antibodies**	**Dilution**	**Suppliers**
Biotinylated horse anti-mouse IgG	1:250	Vector Laboratories Inc., Burlingame, CA, USA
Biotinylated goat anti-rabbit IgG	1:250	Vector Laboratories Inc., Burlingame, CA, USA

## Data Availability

The data presented in this study are available on request from the corresponding author.

## Abbreviations

ABC: avidin-biotin complex; BCCA, bilateral common carotid arteries; BMT, Barnes maze test; CA1, Cornu Ammonis 1 region; CV, cresyl violet; FJB, Fluoro-Jade B; MAP2, microtubule-associated protein 2; MBP, myelin basic protein; NeuN, neuronal nuclei; PAT, Passive avoidance test; ROD, relative optical density; SO, stratum oriens; SP, stratum pyramidale; SR, stratum radiatum; RIP, receptor-interacting protein; TI, transient ischemia; VGLUT-1, vesicular glutamate transporter 1.
